# Supplementary figures and images for: Defining Transcriptomic Heterogeneity between Left and Right Ventricle-Derived Cardiac Fibroblasts
Source: Cells. 2024 Feb 10;13(4):327. doi: 10.3390/cells13040327 (PMC10887120; doi:10.3390/cells13040327)

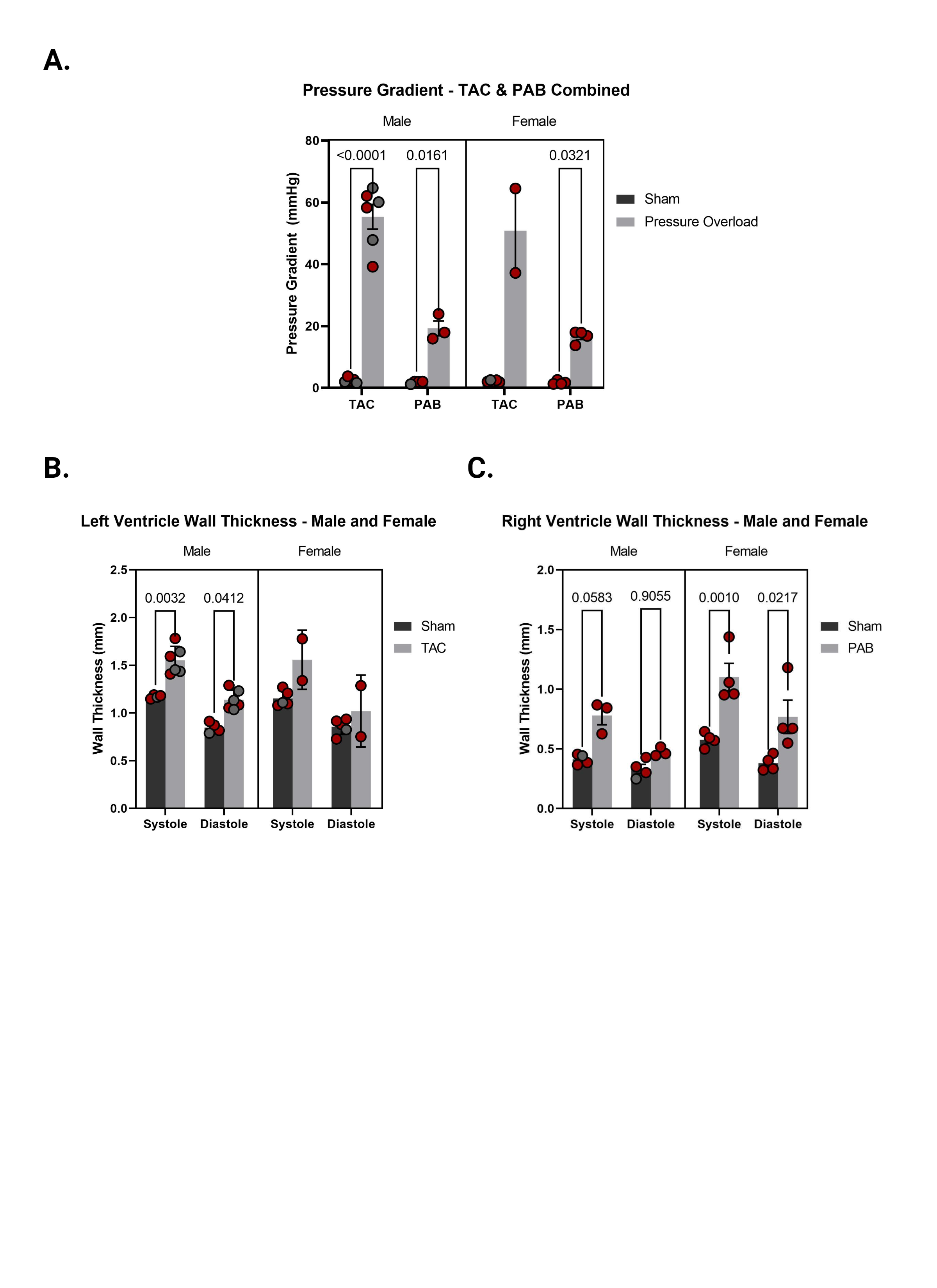

Supplement: Supplementary file 1 [file cells-13-00327-s001.zip › LV vs RV - Supplementary Figure S1.png]

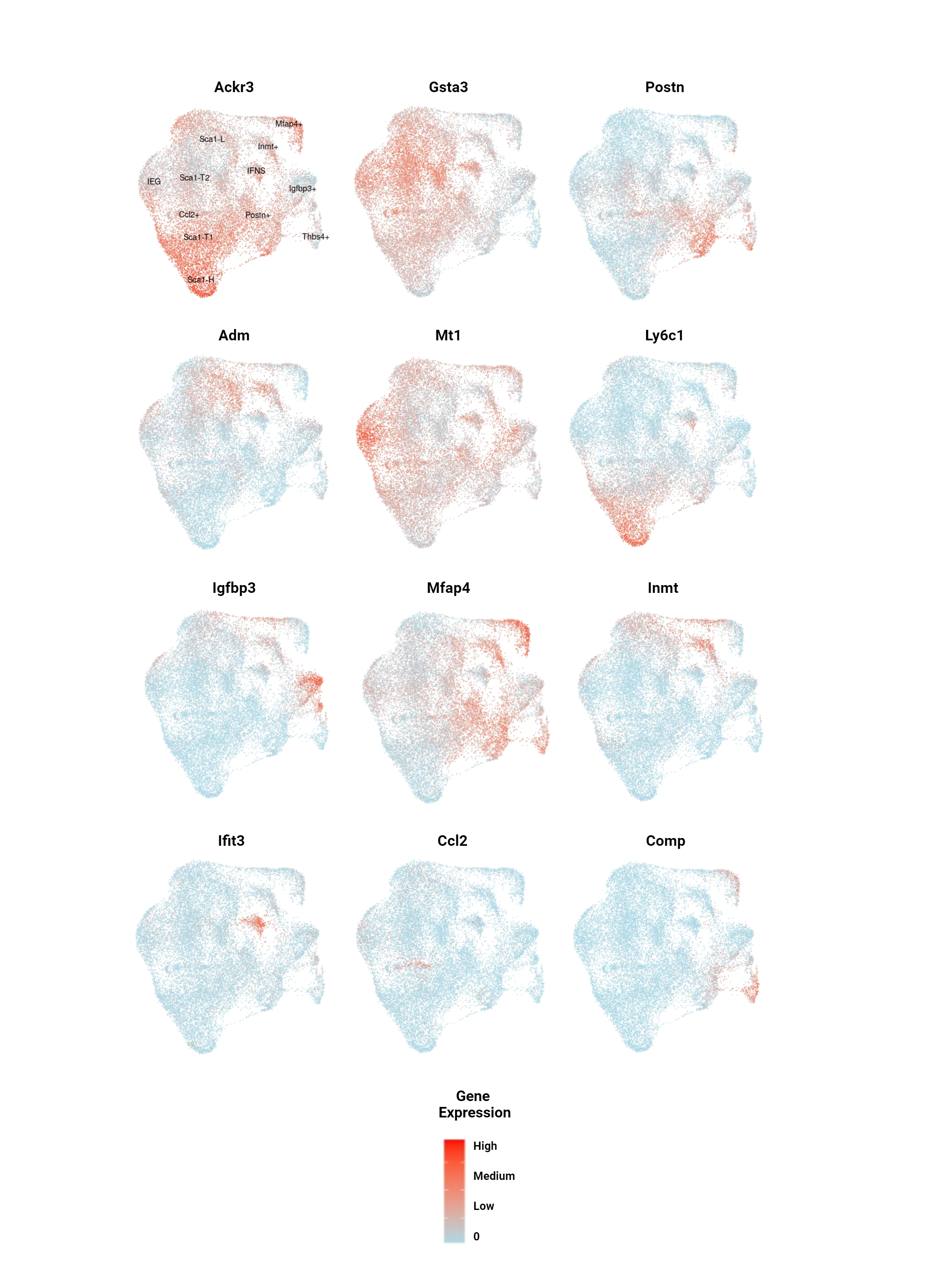

Supplement: Supplementary file 1 [file cells-13-00327-s001.zip › LV vs RV - Supplementary Figure S2.png]

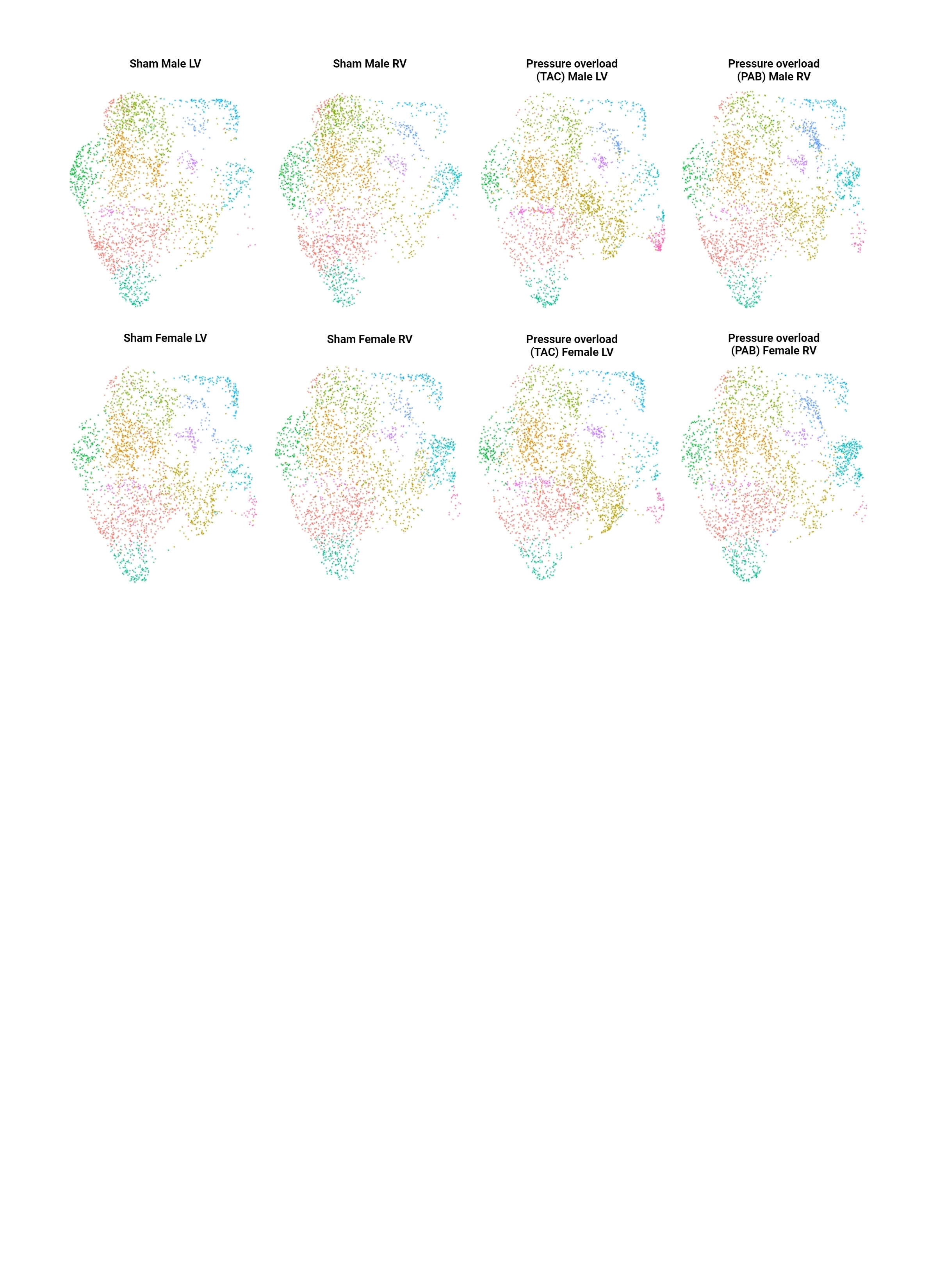

Supplement: Supplementary file 1 [file cells-13-00327-s001.zip › LV vs RV - Supplementary Figure S3.png]

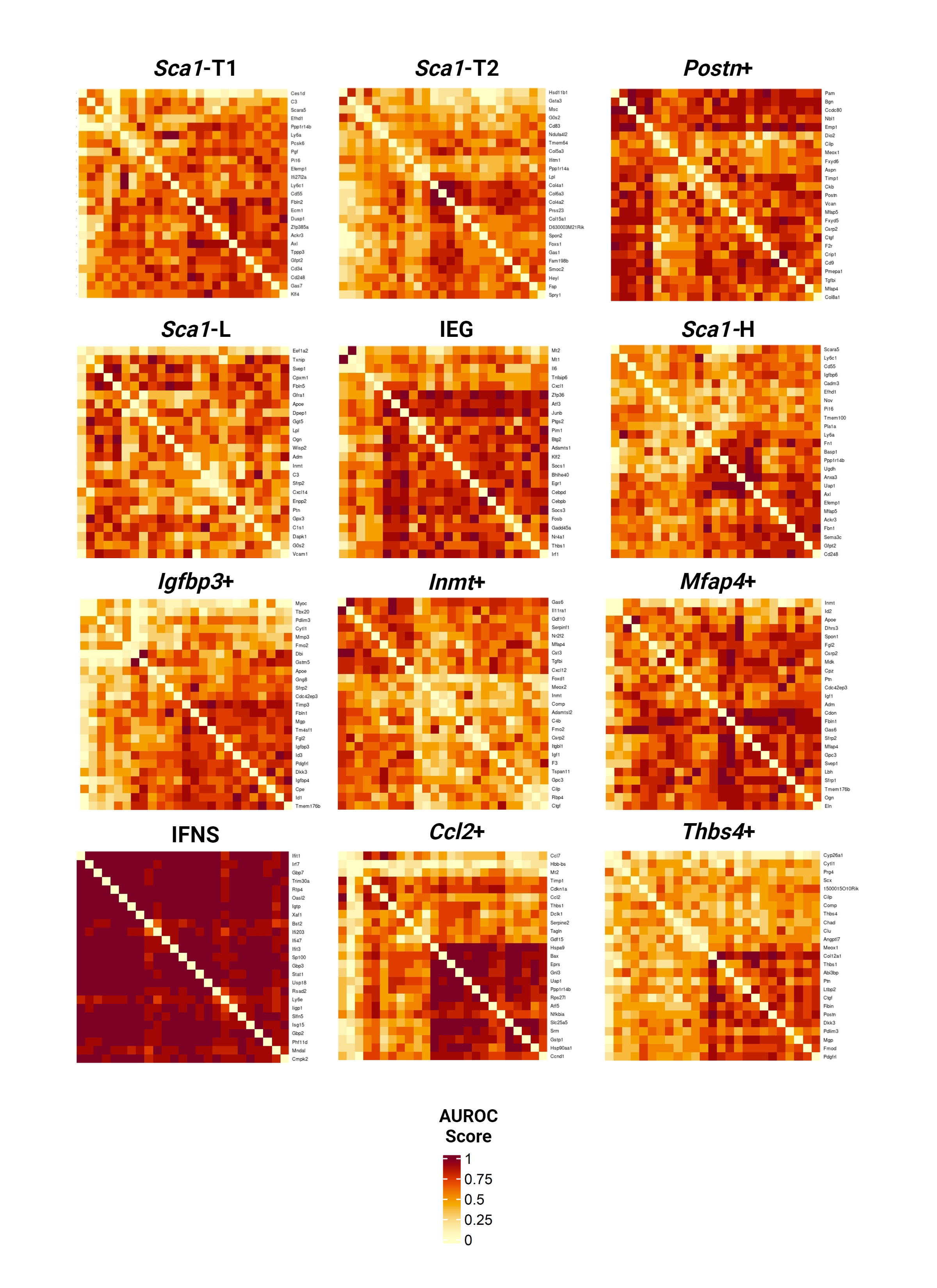

Supplement: Supplementary file 1 [file cells-13-00327-s001.zip › LV vs RV - Supplementary Figure S4.png]
